# Bleaching

**Published:** 1861-10

**Authors:** 


### BLEACHING.

We are glad to see that the subject of bleaching teeth occupied, to a good extent, the attention of the late Convention at New Haven. It is an important and intricate subject; and that it is beginning to elicit the attention of the profession is evidence of progress. We are aware that individuals have given the matter some care; but, as a profession we certainly do not understand the subject.

In looking at this subject, the first inquiry ought to be the chemical characteristics of the coloring materials that are to be decomposed. Without, at least, some general knowledge in this direction, all our efforts will be empirical. Now, it may be sufficient, for our present purpose, to state that most, if not all, of the coloring matters which concern the dentist, are organic compounds. Most of them contain carbon, hydrogen, oxygen and nitrogen. And, it is scarcely necessary to remark that their color is dependent on their composition. If one of their elements be removed, or if their proportion be much varied, the color will be changed or obliterated.

The object to be aimed at in bleaching, then, is to decompose the coloring compound, by some agent or agents for which one or more of its elements have a strong affinity. Of course, the agent of decomposition must have an affinity, for at least one element of the coloring compound, stronger than that by which it is held in the compound.

Bearing these things in mind, we can understand the action of some of the best known bleaching agents.

Sulphurous acid, ($S\ O2$) which is always formed when sulphur is burned, and is commonly called sulphur smoke, has a powerful affinity for oxygen, and usually bleaches by taking that element from the coloring compound. This acid is extensively used by bleachers of straw millinery.

Chlorine, which has been termed "the great bleaching agent," as is well known, has a strong affinity for hydrogen, and sometimes bleaches by taking this element from the coloring compound, and sometimes by taking hydrogen from the water present, thus liberating the oxygen, which in its nascent state, is able to decompose the coloring matter by taking its hydrogen and carbon. In many instances of chlorine bleaching, these processes take place simultaneously, and are not in the least incompatible with each other.

Oxygen, as it exists in the atmosphere, sometimes bleaches, (and sometimes dyes,) by virtue of its affinity for elements or compounds contained in the colored matter.

Cyanide of potassium, as recommended in the September number of the *Cosmos*, by Dr. Kingsbury, bleaches by virtue of the affinity of its cyanogen for hydrogen. Cyanide of potassium is readily decomposed. Its solution undergoes "spontaneous decomposition," even in closed vessels. Its cyanogen, with the energy incident to the nascent state, is able to remove hydrogen from almost any organic compound. Its mode of bleaching is exactly the same as that of chlorine.

Dr K's caution in regard to the poisonous properties of the cyanide are worthy of attention; and the Dr., as well as all others, should know that it bleaches only by forming hydrocyanic acid, the most deadly poison known.

But it is not our intention to write an essay on the subject of bleaching now and here. We set out merely to tell how glad we are that the subject is eliciting increased attention. W.

